# Nonhuman Primate IFITM Proteins Are Potent Inhibitors of HIV and SIV

**DOI:** 10.1371/journal.pone.0156739

**Published:** 2016-06-03

**Authors:** Jordan Wilkins, Yi-Min Zheng, Jingyou Yu, Chen Liang, Shan-Lu Liu

**Affiliations:** 1 Department of Molecular Microbiology and Immunology, Bond Life Sciences Center, University of Missouri, Columbia, Missouri, United States of America; 2 Lady Davis Institute, Jewish General Hospital, Montreal, Quebec, Canada; 3 Departments of Medicine, Microbiology & Immunology, McGill University, Montreal, Quebec, Canada; Emory University School of Medicine, UNITED STATES

## Abstract

Interferon-induced transmembrane (IFITM) proteins are potent antiviral factors shown to restrict the infection of many enveloped viruses, including HIV. Here we report cloning and characterization of a panel of nonhuman primate IFITMs. We show that, similar to human IFITM, nonhuman primate IFITM proteins inhibit HIV and other primate lentiviruses. While some nonhuman primate IFITM proteins are more potent than human counterparts to inhibit HIV-1, they are generally not effective against HIV-2 similar to that of human IFITMs. Notably, depending on SIV strains and also IFITM species tested, nonhuman primate IFITM proteins exhibit distinct activities against SIVs; no correlation was found to support the notion that IFITM proteins are most active in non-natural primate hosts. Consistent with our recent findings for human IFITMs, nonhuman primate IFITM proteins interact with HIV-1 Env and strongly act in viral producer cells to impair viral infectivity and block cell-to-cell transmission. Accordingly, knockdown of primate IFITM3 increases HIV-1 replication in nohuman primate cells. Interestingly, analysis of DNA sequences of human and nonhuman primate IFITMs suggest that IFITM proteins have been undergoing purifying selection, rather than positive selection typical for cellular restriction factors. Overall, our study reveals some new and unexpected features of IFITMs in restricting primate lentiviruses, which enhances our understanding of virus-host interaction and AIDS pathogenesis.

## Introduction

Following detection of pathogen-associated molecular patterns (PAMPs), cells produce and secrete interferon [1, 2]. Interferons are cytokines that upregulate the expression of hundreds of interferon-stimulated genes (ISGs) and represent one of the cells first lines of defense against viruses [3]. Several ISGs have been characterized with antiviral activity, including Tetherin, TRIM5α, APOBEC3G, SAMHD1, and MxB [4–11]. The interferon-induced transmembrane (IFITM) proteins are a subset of ISGs known to restrict several enveloped viruses including, but not limited to, influenza A virus (IAV), dengue virus, Ebola virus, SARS coronavirus, hepatitis C virus (HCV), Jaagsiekte sheep retrovirus (JSRV) and human immunodeficiency virus (HIV) [12–19].

In humans, five IFITM isoforms have been discovered thus far [20]. IFITM5 is found in osteoblasts and functions in bone mineralization while IFITM10 has an unknown function [21, 22]. The remaining three members (IFITM1, 2, and 3) have been characterized with antiviral activity [20, 23]. The IFITM proteins are localized to both the plasma membrane and the endosomal membranes where they are thought to restrict viral entry by directly modulating cell membranes or antagonizing components of the viral structure [19, 24–29]. While the exact topology of IFITMs remains unclear, studies have suggested that the IFITMs have full or partial membrane-spanning regions, with the N-terminus and a conserved central region on the cytosolic side and the C-terminus being extracellular [26, 30, 31]. The N-terminus of human IFITM2 and IFITM3 contain an additional 20 or 21 amino acids, respectively, compared to IFITM1. This extended N-terminal region contains a tyrosine residue (Y20) that is important for IFITM localization and their antiviral activity [32–34]. Recently, the PPxY motif of IFITM3 was discovered to interact with the NEDD4 E3 ligase that causes IFITM3 ubiquitination [35]. Cysteine residues C71, C72, and C105 in IFITM3, which are also conserved in IFITM1 and IFITM2, are palmitoylated and contribute to its antiviral function [24, 31]. Two phenylalanine residues (F75 and F78) in IFITM3 mediate interaction amongst the IFITMs, increasing IFITM3 antiviral properties [36]. Interestingly, IFITM proteins have also been shown to promote infection or replication of some viruses, although the underlying mechanisms remain to be defined [37, 38].

HIV-1 and HIV-2 are the results of zoonotic transmission of SIV into humans from chimpanzee and sooty mangabey, respectively [39, 40]. Adaptation into new hosts often requires viruses to evolve countermeasures to evade new host defenses. For example, TRIM5α from rhesus monkey (TRIM5αrh) is highly effective at restricting HIV-1 [5]. However, TRIM5αrh does not restrict viruses that naturally infect Old World monkeys (SIVmac). These observations were attributed to changes in the sequence of viral capsid from SIV to HIV [5]. Similarly, human Tetherin is less effective restricting HIV when compared to that of SIV and vice versa [41]. The decreased inhibitory effects of human TRIM5α and Tetherin on HIV-1 account for efficient spread and pathogenesis of HIV-1 in humans [42].

Canonically, restriction factors are characterized by their ability to restrict viruses in non-natural hosts and have undergone positive selection due to viral antagonism. We find here that while nonhuman primate IFITM proteins are in general more potent to restrict HIV-1, they are still highly effective against some strains of SIVs derived from their own natural hosts. Moreover, phylogenetic analysis shows that primate IFITM proteins have been undergoing purifying selection, suggesting that the primary amino acid sequences of IFITMs are essential for their structure and functions, which is atypical for restriction factors.

## Materials and Methods

### Cells, plasmids, and reagents

SupT1 (NIH AIDS Reagent Program) and Jurkat-inGLuc (gift from Walther Mothes) cells were maintained in Roswell Park Memorial Institute medium (RPMI-1640) with 10% fetal bovine serum (FBS) and 1% penicillin-streptomycin. HeLa-TZM-bl (NIH AIDS Reagent Program) and HEK293T (ATCC) cells were maintained in Dulbecco’s modified Eagle’s medium (DMEM) with 10% FBS and 1% penicillin-streptomycin. Nonhuman primate fibroblasts were obtained from Coriell Institute cell repositories (Camden, NJ). Fibroblasts were either maintained in DMEM and/or Minimum Essential Medium (MEM) with 10–15% FBS and 1% penicillin-streptomycin according to Coriell’s specimen specifications. All cell lines were incubated in a 5% CO2 atmosphere at 37˚C.

All IFITMs were cloned into the retroviral vector pQCXIP containing an N-terminal Flag tag. The following proviral DNAs were obtained from NIH AIDS Reagent Program: HIV-1-NL4.3 (#114), HIV-1 BH10 (#90), HIV-2-ROD1 (#207), SIVcpzTAN2.69 (#11497), SIVagmTAN (#3444), and SIVmac239 (#210). The pCMV-BlaM-Vpr construct was purchased from Addgene (#21950) (described in [43]). Lentiviral vectors containing control-shRNA or IFITM3-shRNA were purchased from Sigma. The anti-FLAG beads (F2426), anti-FLAG (F3165) and anti-β-tubulin (T8328) antibodies were purchased from Sigma. Anti-IFITM3 antibody was purchased from Proteintech (11714-1-AP). The following antibodies were obtained from NIH AIDS Reagent Program: anti-HIV-1 gp120 (#288), anti-HIV-1 gp41 (#1475), and anti-HIV-1 p24 (#1513).

### Cloning and DNA sequencing

Nonhuman primate IFITM genes were obtained from the following primate cell lines (Coriell Institute, Camdem, NJ): gorillia (*Gorilla gorilla gorilla*), L’Hoest’s monkey (*Cercopithecus lhoesti*), moustached monkey (*Cercopithecus cephus cephus*), red-capped mangabey (*Cercocebus torquatus torquatus*), mandrill (*Mandrillus sphinx*), Syke’s monkey (*Cercopithecus albogularis*), and guereza (*Colobus guereza*). Briefly, the total RNAs were extracted from these non-human primate cell lines using an RNeasy kit (Qiagen). Reverse transcription was carried out by using 1μg of total RNA, plus random primer (Thermal Fisher Scientific) and Superscript III (Invitrogen) following manufacturer’s instructions. IFITM genes were PCR amplified from cDNAs using primers IFITM1 forward/reverse (CAACAGGGGAAAGCAGGGCTC/ CTGTATCTAGGGGCAGGACCAAG) and IFITM3 forward/reverse (CAACACTTCTTTCCCCAAAGCCAG/ TTGTGGACAGGTGTGTGGG). PCR products were cloned into PCR2.1 vector, and 3–5 clones from each species were sequenced. A Flag tag was introduced to the N termini of primate IFITMs by PCR, and the insert IFITM genes in PCR2.1 were subcloned into pQCXIP vector at BamHI and EcoRI sites. All IFITM genes on pQCXIP vector were confirmed by DNA sequencing.

### Stable cell lines

SupT1 and Jurkat-inGLuc cell lines stably expressing IFITMs were produced by transduction of cells with pQCXIP-IFITM constructs [19, 29]. Briefly, HEK293T cells were seeded in 35 mm dishes and transfected with 1 μg pQCXIP-flag-IFITM, 1 μg pCMV-gag-pol-MLV, and 0.1 μg pVSV-G using calcium phosphate method. Viral supernatants were harvested over 72 hours and clarified via centrifugation. Retroviral particles were used to infect SupT1 or Jurkat-inGLuc cells in the presence of 4 μg/ml polybrene by spinoculation at 1,850 x g for 1 hour at 4˚C. Stably transduced cells were selected for with 1 μg/ml puromycin.

To knockdown IFITM3 in gorilla fibroblasts (Cat #), HEK293T cells were cotransfected with pHIV-Δ8.2-Gag-Pol, lentiviral shRNA (Sigma), and VSV-G. Produced virions were used to infect gorilla fibroblast. Transduced fibroblasts were selected for with 1 μg/ml puromycin.

### Viral replication assay

For HIV-1 long-term replication, 5 X 10^5^ SupT1 cells were challenged with appropriate volumes of the wild type NL4.3 viral stock; unbound virus was removed 6 hours after the initiation of infection and cells were incubated with fresh RPMI-1640 medium. Supernatants containing newly produced virions were collected every 2 days until a complete cell death was observed. Supernatants were measured for viral production by performing reverse transcriptase (RT) assay (see below) and for infectivity by infecting HeLa-TZM-bl cells.

### RT assay

The experiments were performed as previously described [15]. Briefly, 10 μL virions containing culture supernatants were incubated with 40 μL of reaction mixture at 37°C for 3 h. The mixture contains 50 mM Tris-HCl pH 7.9, 5 mM MgCl2, 0.5 mM EGTA, 0.05% Triton X-100, 2% (V/V) ethylene glycol, 150 mM KCL, 5 mM DTT, 0.3 mM GSH (reduced glutathione), 0.5 U/mL poly (rA) oligo (dT), and 0.1 μCi/μL 3H dTTP (Perkin-Elmer). The reaction was stopped by adding 10% (V/V) cold Trichloroacetic Acid (TCA) at 4°C for 30 min, and the mixture was transferred to Millipore MultiScreen Glass Fiber FC plate. After being washed twice with cold 10% (V/V) TCA and cold ethanol, the membranes were inserted into Beta Gamma vials and read in Microbeta counter (Beckman Coulter).

### Infectivity assay

SupT1 cells were seeded at 4 X10^5^ cells/ml in 12-well dishes. Infectious virus was added in the presence of 4 μg/ml polybrene with spinoculation at 1,850 X g for 1 hour at 4˚C and then incubated at 37˚C for 6 hours. SupT1 cells were then refed and incubated for an additional 48 hours. Viral supernatants were used to infect HeLa-TZM-bl cells. Forty-eight hours post-infection, HeLa-TZM-bl cells were harvested in lysis buffer (50 mM Tris pH 7.4, 150 mM NaCl, 1 mM EDTA, 1 mM EGTA, 1% Triton X-100). HeLa-TZM-bl lysates were mixed at a 1:1 ratio with assay buffer (200 mM Tris pH 7.8, 250 mM MgCl2, 7.5 mM ATP, 25 mM CoA, 0.2 mg/ml D-Luciferin) and measured for luciferase activity using a Perkin Elmer plate reader.

### Immunoprecipitation and western blot

HEK293T cells were cotransfected with HIV-1-NL4.3 and increasing amounts of IFITM plasmids. pQCXIP-empty was used as a control and to ensure all transfections had an equivalent amount of total plasmid during transfection. At 48 hours post-transfection, HEK293T cells were harvested in RIPA buffer (containing 0.05% SDS). Cell debris was removed by centrifugation. A fraction of the lysate was used for total input analysis by Western blot while the rest was subjected to immunoprecipitation using anti-FLAG beads. Immunoprecipitations were incubated overnight at 4˚C with rotation. Beads were centrifuged and washed three times with cold lysis buffer. All samples were boiled in sample buffer prior to resolving by SDS-PAGE. The gels were transferred to polyvinylidene difluoride (PVDF) membranes and blocked in blocking solution (5% non-fat milk in PBS 0.1% Tween-20) for 1 hour at room temperature. Membranes were washed (PBS with 0.1% Tween-20) three times followed by overnight incubation with the primary antibody in blocking solution overnight at 4˚C. The membranes were washed three times and incubated with secondary antibodies conjugated to HRP for 1 hour at room temperature. The blots were incubated with HRP substrate (Millipore # WBLUR0100) and bands were visualized using a Fuji Imager.

### Virion fusion assay and flow cytometry

The fusion assay was performed as previously described [43]. HEK293T cells were seeded onto 60 mm plates and cotransfected with 3 μg of HIV-1-NL4.3 and 1 μg of pCMV-BlaM-Vpr; virions were harvested 48–72 h post-transfection. SupT1 cells were incubated with virus and spinoculated at 4˚C for 1 hour and then further incubated at 37˚C for 3 hours. SupT1 cells were resuspended in CO2-independent media and loaded with CCF2 dye according to manufacturer’s recommendations (Invitrogen). Cells were washed and incubated in CO2-independent media with 10% FBS and 0.5 μM probenecid for 16 hours at room temperature. Samples were fixed in 2% formaldehyde, washed, and resuspended in PBS. Uncleaved CCF2-AM dye was detected at Ex409/Em518 and cleaved CCF2 was detected at Ex409/Em447 using a BD Fortessa flow cytometer.

### Cell-to-cell infection

For cell-to-cell infection, we followed a system previously described in [29]. Briefly, Jurkat-inGLuc cells, expressing IFITMs or not expressing IFITMs, were spinoculated at 1,850 X g with infectious virus for 1 hour at 4˚C followed by incubation at 37˚C for 4 hours. Cells were then treated with 10 nM phorbol myristate acetate (PMA) for 4 hours. The Jurkat-inGLuc cells were then washed and refed for 18 hours at 37˚C. The infected Jurkat-inGLuc donor cells were split in half for either cell-free or cell-to-cell infection. For cell-free infection, Jurkat-inGLuc cells were incubated for 48 hours at 37˚C. Newly generated virus was used to infect SupT1 target cells for 48 hours. Media from SupT1 target cells was assayed for guassia Luciferase activity. For cell-to-cell infection, Jurkat-inGLuc cells were incubated with an equivalent number of SupT1 cells expressing or not expressing IFITMs. At 48 hours post-coculture, supernatants were assayed for guassia Luciferase activity using a Perkin Elmer plate reader.

### DNA sequence alignment and selection analysis

For this study, 14 new nonhuman primate IFITM sequences were used (see under Cloning and DNA Sequencing), among which 13 are unique sequences (6 IFITM1 and 7 IFITM3) at the nucleotide level and 11 are unique sequences (4 IFITM1 and 7 IFITM3) at the amino acid level. A total of 18 nucleotide sequences were used for IFITM analysis within Datamonkey online interface (http://www.datamonkey.org/dataupload.php) and MEGA6 software [44]. Sequences were aligned using ClustalW and a phylogeny of IFITM was constructed by the Neighbor-joining method [45, 46]. Evolutionary distances were estimated using the Jukes Cantor method [47]. Confidence levels were determined using the bootstrap method with 1000 replicates and selection was determined using the Nei-Gojobori method [48].

### GenBank accession numbers

The GenBank accession numbers for the cloned nonhuman primate IFITMs are KU570002-KU570015.

## Results

### Nonhuman primate IFITMs differentially restrict HIV

**Fig 1:**
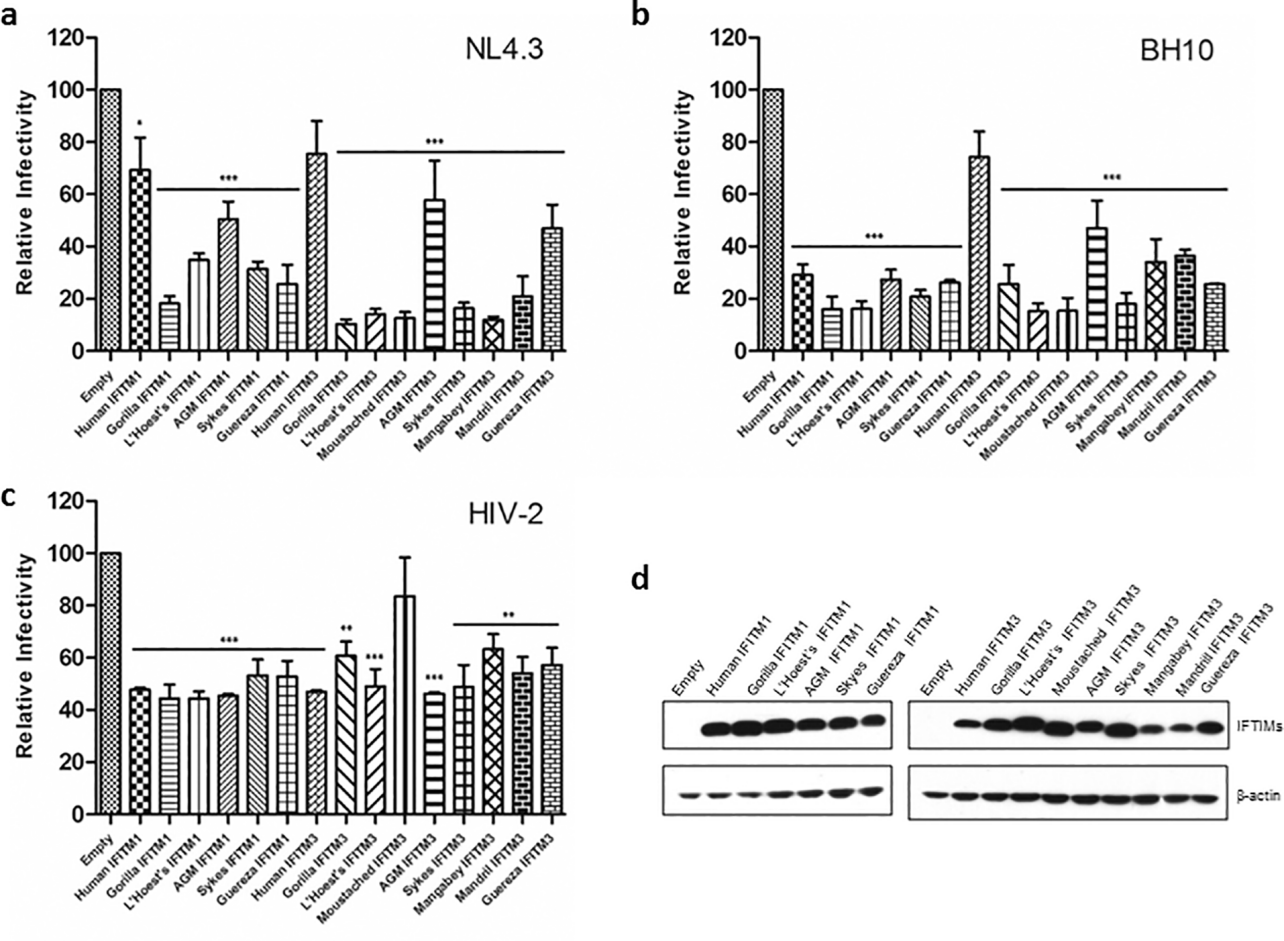
Nonhuman primate IFITM proteins restrict HIV-1 and HIV-2. Stably transduced SupT1 cells not expressing or expressing IFITM proteins were infected with the indicated starins of HIV pseudotyped by VSV-G. At 48 hours post-infection, the infectivity of newly generated infectious viral particles was determined by infecting the indicator cell line HeLa-TZM-bl. Relative infectivity to the empty vector control is shown. Standard errors were determined from 3–7 independent experiments (* indicates a p-value < 0.05). The expressions of IFITM proteins in SupT1 cells are shown in (D).

HIV has adapted various mechanisms to escape host restriction [42]. While it was previously shown that IFITMs from African green monkey (AGM) are capable of restricting both HIV-1 and SIVagm to varying degrees [49], we further sought to know if the IFITMs from additional nonhuman primates are capable of restricting HIV. To do so, we cloned IFITM1 and IFITM3 from 8 nonhuman primate cell lines belonging to the families *Cercopithecidae* (old world monkey; OWM) and *Hominidae* (hominoids), stably expressed them in the CD4+ T cell line SupT1, and tested their ability to restrict HIV (Table 1, Fig 1). SupT1 cells were infected with HIV-1 (NL4.3 or BH10) or HIV-2 bearing VSV-G in order to achieve efficient infection, and newly produced virus was harvested 48 hours later. Infectivity of newly generated virus was measured using the indicator cell line HeLa-TZM-bl (TZM-bl), which is susceptible to both CXCR4 and CCR5 tropic viruses. Against HIV-1 NL4.3, we found that both human IFITM1 and IFITM3 had less than a two-fold reduction in infectivity, similar to our previous results [50], while we found up to an approximate 5- and 10-fold reduction in infectivity for the majority of nonhuman primate IFITM1 and IFITM3 tested (Fig 1A). Against HIV-1-BH10, human IFITM1 resulted in an approximate 4-fold reduction in infectivity while human IFITM3 caused less than a 2-fold reduction (Fig 1B), again paralleling our previous reports [15, 51]. Notably, nonhuman primate IFITM1 and IFITM3 reduced HIV-1 BH10 infectivity up to 10- and 6-fold, respectively (Fig 1B). Interestingly, against HIV-2, we found only a two-fold reduction in infectivity against almost all species of IFITMs (Fig 1C). Similar to human IFITMs [29, 32], nonhuman primate IFITMs did not significantly inhibit release of HIV-1 and HIV-2 from viral producer cells based on their RT activities (data not shown). Western blotting analysis showed that the expression levels of these nonhuman primate IFITMs were approximately comparable to their human counterparts (Fig 1D). Altogether, these results suggest that HIV-1 is strongly, but differentially, restricted by IFITMs of nonhuman primate species.

**Table 1:** Primate IFITMs used in this study.

Family^a^	Taxonomic^b^	Common name^c^
Hominidae	Homo sapiens	Human
Hominidae	Gorilla	Western lowland gorilla
Cercopithecidae	Cercopithecus lhoesti	L'Hoest's monkey
Cercopithecidae	Cercopithecus cephus	Red-tailed moustached monkey
Cercopithecidae	Cercocebus torquatus	Red-capped mangabey
Cercopithecidae	Mandrillus sphinx	Mandrill
Cercopithecidae	Cercopithecus albogularis	Syke's monkey
Cercopithecidae	Colobus guereza	Guereza
Cercopithecidae	Chlorocebus aethiops	African green monkey

^a^ Family name (Hominidae or Cercopithecidae)

^b^ Organismal classification

^c^ Common name associated with each primate

### Nonhuman primate IFITMs restrict different strains of SIV

**Fig 2:**
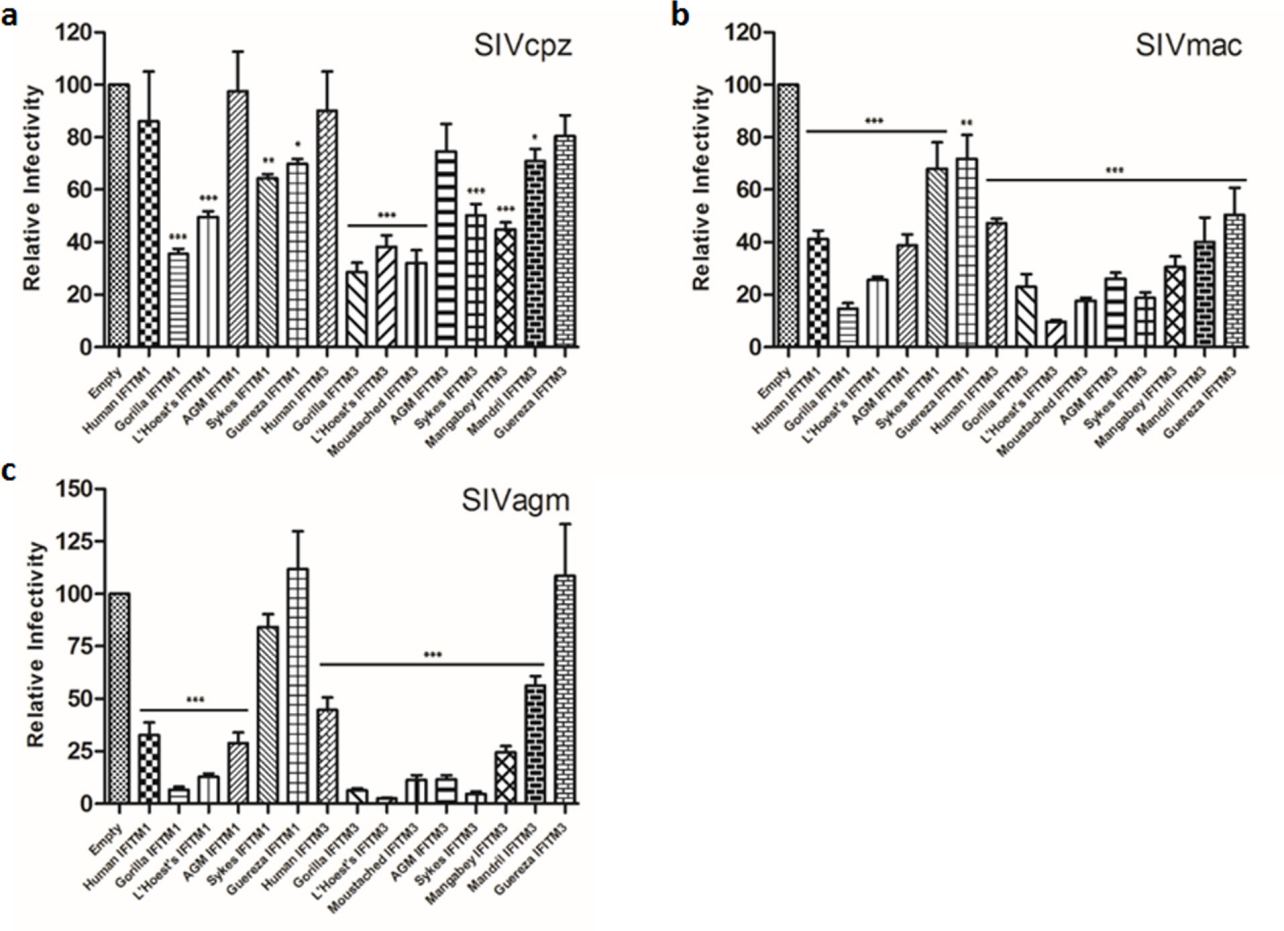
Nonhuman primate IFITM proteins restrict SIVs. SupT1 cells not expressing or stably expressing IFITM proteins were infected with the indicated SIV strains bearing VSV-G. At 48 hours post-infection, the infectivity of newly generated infectious viral particles was determined by infecting HeLa-TZM-bl indicator cells. Relative infectivity to the empty vector control is shown. Standard errors were determined from 3–10 independent experiments (* indicates a p-value < 0.05).

HIV-1 resulted from zoonotic transmission of SIVcpz from chimpanzee (*Pan troglydytes*; CPZ) to humans while HIV-2 arose from SIVsm of sooty mangabey (*Cercocebus atys*; SM) [39, 40, 52]. We therefore tested whether or not nonhuman primate IFITMs could also restrict different strains of SIV in SupT1 cells stably expressing individual IFITM proteins. We found that, while some nonhuman primate IFITM1 and IFITM3 (such as Gorilla, L'Hoest's and Moustached) caused an approximate 2 to 3-fold reduction in infectivity of SIVcpz, others showed activity similar to that of humans (less than 2-fold) (Fig 2A). Against SIVmac, human IFITM1 and IFITM3 caused roughly a 2-fold reduction in infectivity while nonhuman primate IFITM1 and IFITM3 resulted in up to a 7- and 10-fold reduction, respectively (Fig 2B). The most striking differences between human and nonhuman primate IFITM restriction of SIV was found against SIVagm. While human IFITMs caused an approximate 2- to 3-fold reduction in infectivity, nonhuman primate IFITM1 and IFITM3 restricted SIVagm by up to15 to 40-fold in infectivity (Fig 2C). Of note, the antiviral activities of IFITM1 derived from Sykes and Guereza, as well as IFITM3 derived from Mandrill, Guereza, and Mangabey, were much weaker against both SIVmac and SIVagm than IFITMs of other species, again highlighting the species-dependent antiviral activities of IFITMs.

On the basis of the degrees of inhibition, we ranked the ability of nonhuman primate IFITMs to inhibit the primate lentiviruses as SIVagm > SIVmac, HIV-1 BH10, HIV-1 NL4.3 > SIVcpz > HIV-2. Overall, IFITMs derived from Gorilla, L'Hoest's, Moustached, and Sykes exhibit stronger inhibition as compared to those from Mandrill, Guereza, and Mangabey. These results collectively suggest that HIV/SIV restriction is conserved across primate IFITMs, yet in a highly virus-strain and IFITM-species dependent manner.

### Nonhuman primate IFITM proteins interact with HIV envelope

**Fig 3:**
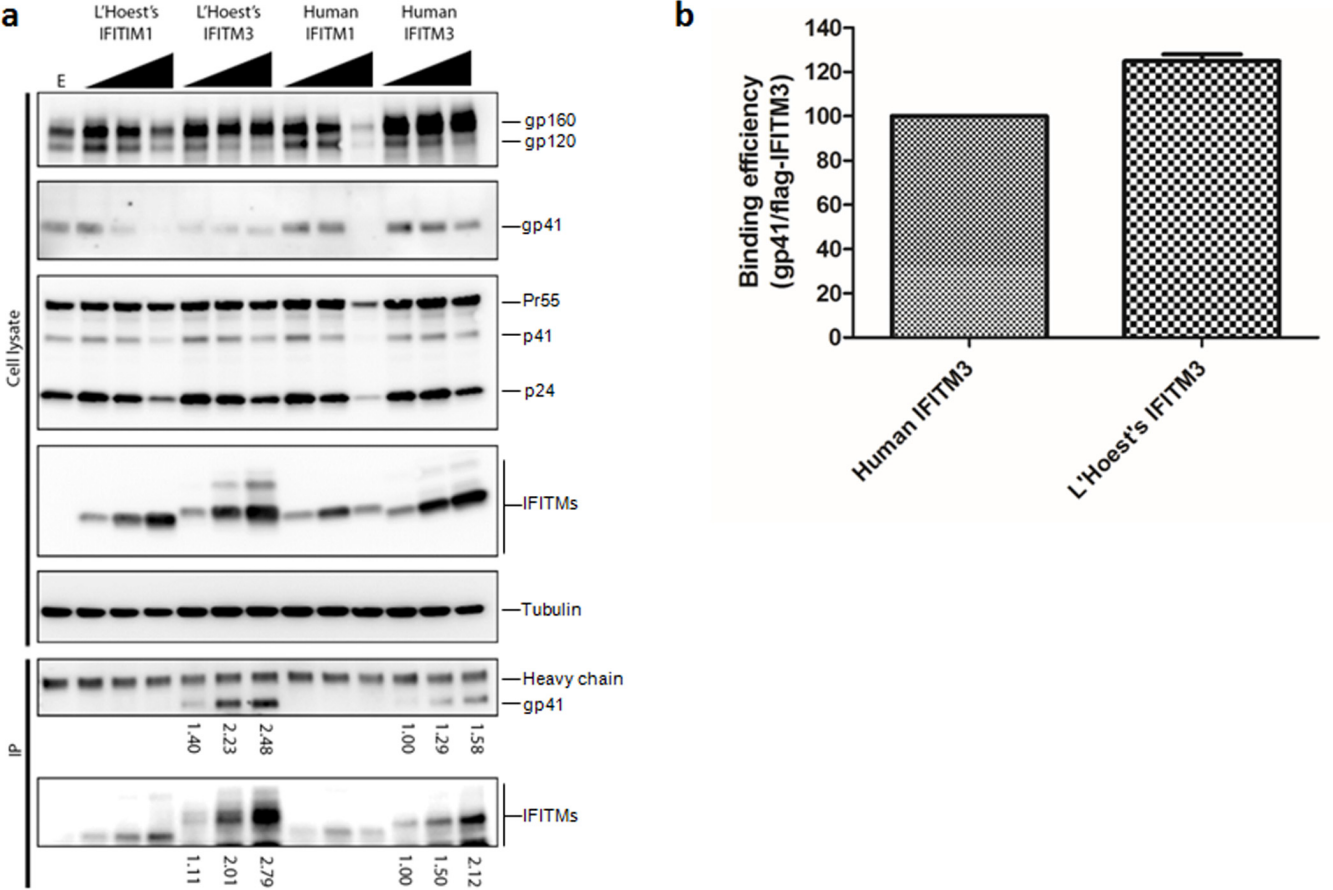
Nonhuman primate IFITM3 interacts with HIV-1 envelope. 293T cells were transfected with expression plasmids encoding HIV-1 NL4.3 without or with increasing amounts of the indicated IFITM plasmids. At 48 hours post-transfection, 293T cells were harvested in RIPA buffer and clarified of cell debris. Flag-IFITM was immunoprecipitated from 293T lysates using anti-Flag-beads. Immunoprecipitated products were washed three times and harvested in sample buffer. (**a**) Cell lysates and immunoprecipitated products were detected via Western blot using indicated antibodies. (**b**) The band intensities for immunoprecipitated gp41 and flag-IFITM bands were measured using Multigauge software (shown below each band). The averaged ratio of gp41/flag-IFITM is shown (confirmed from two independent experiments). About a 20% increase in binding efficiency for L’Hoest’s IFITM3 with gp41 is seen when compared to that of human IFITM3. Measurements for IFITM1 were not determined due to lack of detection of coimmunoprecipitated gp41.

Recently, our lab demonstrated that human IFITM proteins contribute to HIV-1 restriction by antagonizing envelope protein (Env), thereby impairing viral fusion, infectivity and cell-to-cell infection [29]. We sought to ask if association of human IFITM with HIV-1 Env is conserved in OWM IFITMs. Because of its strong inhibition of HIV-1 infectivity (Fig 1), we chose L'Hoest's IFITMs to represent OWM. We co-transfected 293T cells with HIV-1 NL4.3 and flag-tagged human or L'Hoest's IFITMs, followed by immunoprecipitation using anti-flag beads. Both human and OWM IFITM3 proteins were capable of interacting with HIV-1 Env in a dose-dependent manner, showing that IFITM-envelope interaction has been evolutionarily conserved (Fig 3A). When we compared the band intensity ratios between immunoprecipitated HIV-1 Env gp41 and flag-IFITM3, we found an approximate 20% increase in OWM IFITM3 interaction with HIV-1 Env compared to that with human IFITM3 (Fig 3B); this was despite the relatively low levels of Env expression in the OWM IFITM3-expressing cells as compared to cells expressing human IFITM3 (Fig 3A; see gp120 and gp41 blots). Human IFITM1 had no significant interaction HIV-1 Env (Fig 3A). Both human and OWM IFITM3 proteins inhibited the processing of Env precursor into gp120 and gp41 (Fig 3A, top panel), whereas the IFITM1 protein downregulated the expression of HIV-1 Env and Gag at higher doses of transfection (Fig 3A; see gp41 and p24 blots) similar to our recent study [29]. These results demonstrate that Env interaction with IFITMs, as well as their effects on Env processing, are evolutionarily conserved among human and nonhuman primates species and that the increased interaction between L'Hoest's IFITM3 and HIV-1 Env somewhat correlates with the enhanced antiviral effect.

### Nonhuman primate IFITM proteins inhibit virion-cell fusion of HIV-1

**Fig 4:**
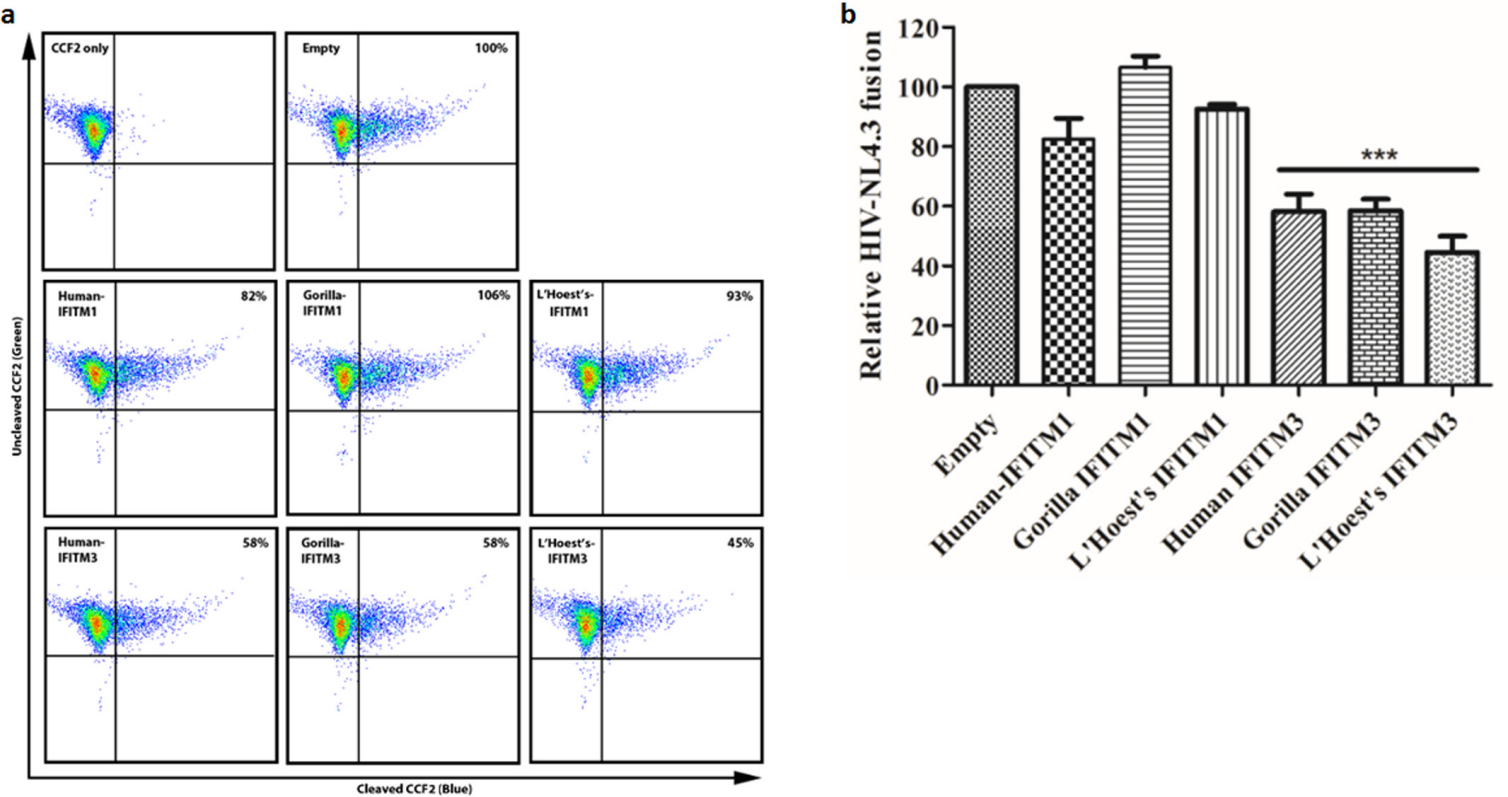
Nonhuman primate IFITM proteins restrict HIV-1 entry. Stably transduced SupT1 cells expressing the indicated IFITM proteins were infected with HIV-1 NL4.3-BlaM-Vpr for two hours. Cells were then loaded with the BlaM substrate CCF2. (**a**) The percent of successful HIV-1 NL4.3-BlaM-Vpr entries (cleaved CCF2) is shown relative to SupT1-Empty control. (**b**) Relative entry of HIV-1 NL4.3-BlaM-Vpr is summarized from three independent experiments.

Entry of HIV-1 has previously been shown to be restricted by human IFITM proteins in target cells [15, 53]. We asked if this function was conserved in OWM IFITM. To analyze entry of HIV-1 in SupT1 cells stably expressing various primate IFITMs, we utilized the ß-lactamase-Vpr (BlaM-Vpr) virion fusion assay described in [43]. HIV-1 virions were generated by cotransfecting HEK293T cells with BlaM-Vpr and HIV-1-NL4.3. Purified virus particles were used to infect SupT1 cells loaded with the BlaM-specific fluorescent substrate CCF2. Successful HIV-1 virion entry was detected by measuring BlaM cleavage of CCF2, which shifts CCF2 fluorescence emission from 520 nm (green) to 447 nm (blue) (Fig 4A). The virion fusion assay results demonstrate that the ability of IFITM to restrict HIV-1 entry is conserved in nonhuman primate IFITMs, particularly IFITM3 (Fig 4A and 4B).

### Nonhuman primate IFITM proteins potently restrict HIV cell-to-cell spread and replication

**Fig 5:**
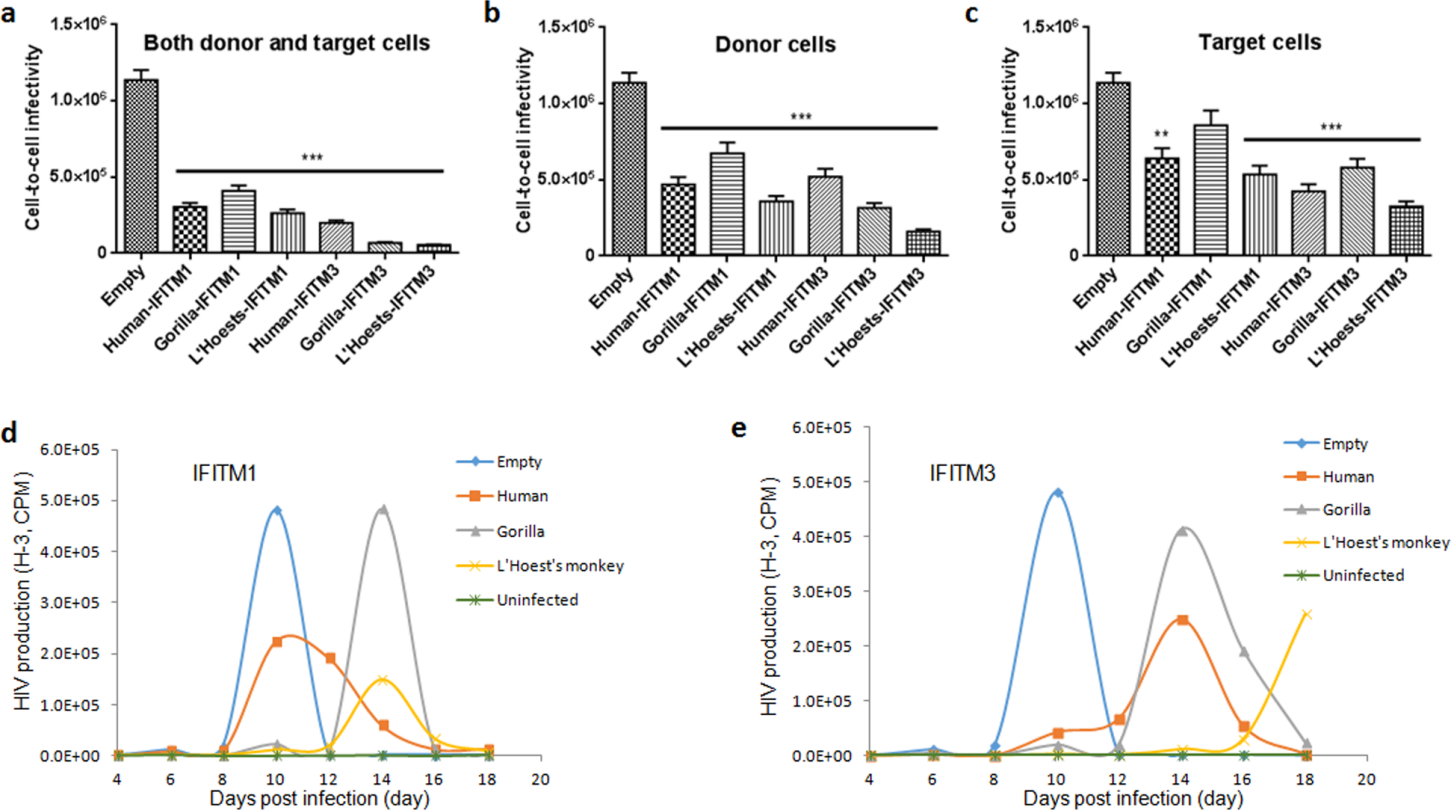
Nonhuman primate IFITM proteins restrict cell-to-cell transmission. (**a, b** and **c**) For cell-to-cell infectivity studies, Jurkat-F3-gLuc donor cells stably expressing or not expressing IFITM were infected with HIV-1 NL4.3-VSV-G. Infected Jurkat cells were cocultured with SupT1 cells stably expressing or not expressing IFITM for 48 hours. Supernatants containing guassia luciferase were assayed for activity as a measure of cell-to-cell infectivity. Average means and standard errors were determined from three independent experiments (* indicates a p-value < 0.05). (**d** and **e**) Prolonged HIV-1 replication in SupT1 cells expressing different IFITM1 or IFITM3. Supernatants were harvested every 2 days until cell death occurred, and RT activities were measured at each time point.

Human IFITM proteins have previously been reported to restrict HIV cell-to-cell spread and replication [28, 29]. We next asked if there were any differences between primate IFITM restriction of cell-to-cell spread of HIV-1 (Fig 5). This was important, because HIV-1 entry appeared not to be dramatically affected by the various IFITMs tested (Fig 4). We utilized a method previously described in [54] that relies on an intron-regulated guassia luciferase (inGLuc) reporter gene that is expressed upon successful HIV infection of target cells [55]. Note that this assay measures only one-round of HIV-1 infection because it is based on an HIV-1 NL4.3 lentiviral vector. Donor Jurkat-inGLuc cells, either expressing or not expressing IFITMs, were infected with HIV-1 NL4.3 bearing VSV-G; 12 h post-infection, half of the cells were used to produce virus for cell-free infection while the other half were used for co-culture with target SupT1 cells expressing or not expressing IFITMs. Human IFITMs showed the greatest restriction (7-fold decrease for IFITM3) of HIV-1 cell-to-cell spread when human IFITM was expressed in both the target and donor cells (Fig 5A–5C) [28, 29, 53]. Notably, while a similar trend was seen for nonhuman primate IFITMs (Fig 5A–5C), there was a 21-fold decrease in HIV-1 cell-to-cell spread when both donor and target cells expressed OWM IFITM3 (3-fold greater compared to human IFITM3) (Fig 5A). These results show that nonhuman primate IFITMs share similar, perhaps modestly enhanced, cell-to-cell restriction of HIV-1 infection as those of human IFITMs.

We next examined the effects of primate IFITMs on HIV-1 replication by performing long-term replication assays using infectious WT NL4.3. Supernatants from cell culture were harvested every two days and quantified for viral production using reverse transcriptase (RT) assay. As shown in Fig 5D, the NL4.3 replication peaked on day 10 in both empty vector and human IFITM1-expressing SupT1 cells, whereas two nonhuman primate IFITM1 delayed the peak to day 14. Of note, human IFITM1 exhibited roughly half the viral production of empty vector control, consistent with the notion that its inhibitory effect on HIV-1 NL4.3 replication is modest [29]. We also found that the L’Hoest’s OWM IFITM1 had a lower peak value compared to the gorilla counterpart (Fig 5D). When IFITM3 protein was compared, we observed a peak shift of viral replication from day 10 to day 14 for both human and gorilla IFITM3 (Fig 5E). Strikingly, HIV-1 replication in L’Hoest’s OWM IFITM3-expressing cells did not peak even after day 18 (Fig 5E). Overall, our results demonstrate that IFITM inhibition of HIV-1 replication is evolutionarily conserved, and that nonhuman primate IFITMs are more potent to inhibit HIV-1 replication than that those of human counterparts.

### Knockdown of endogenous IFITM3 in gorilla fibroblasts promotes HIV-1 replication

**Fig 6:**
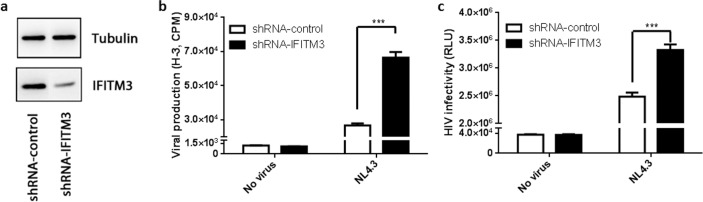
Knockdown of IFITM3 in gorilla fibroblasts decreases HIV-1 restriction. (**a**) Gorilla fibroblasts were transduced with a lentiviral vector containing a small hairpin RNA (shRNA) against IFITM3 or a control shRNA (non-specific). Knockdown efficiency of IFITM3 was determined by Western blot. Tubulin was used as a loading control and IFITM3 was detected using αnti-IFITM3. (**b** and **c**) Gorilla fibroblasts containing shRNAs were infected with HIV-1 NL4.3-VSV-G. At 48 hours post-infection, newly produced viral supernatants were measured for infectious particles by infecting HeLa-TZM-bl indicator cells (**b**) or measured for reverse transcriptase activity (**c**). Averages and standard errors were determined from three independent experiments (* indicates a p-value < 0.05).

To ensure that endogenous IFITM3 of nonhuman primate cell origin contributed to the restriction of HIV-1, we knocked down IFITM3 in gorilla fibroblasts and examined the effect on HIV-1 short-term replication (Fig 6). Gorilla IFITM3 was knocked down using a lentiviral small hairpin RNA against IFITM3. Expression of gorilla IFITM3 was reduced compared to that of a non-specific control shRNA (Fig 6A). Gorilla cells were infected with HIV-1 NL4.3 psuedotyped with VSV-G to ensure entry. After a single round of replication, supernatants containing newly generated virus were harvested and examined for RT activity and by infecting HeLa-TZM-bl cells (Fig 6B and 6C, respectively). There was more than a 2-fold increase in RT activity of viral supernatants from IFITM3 knockdown cells (Fig 6B). Consistently, the infectivity of produced virus was also increased when gorilla IFITM3 was knocked down (Fig 6C). These results support the conclusion that the ability of endogenous nonhuman primate IFITMs to restrict lentiviruses is ancestrally conserved.

### IFITM proteins are under purifying selection

Analysis of protein-coding nucleotide substitutions can provide insight into the type of selective pressure on proteins over time [56]. Nucleotide substitutions that result in a change in the amino acid sequence are referred to as nonsynonymous (dN) while silent changes are considered synonymous (dS). A ratio of nonsynonymous over synonymous substitutions (dN/dS) can be used to detect for positive, neutral, or purifying selection (also referred to as negative selection) [56]. To infer what type of selective pressure the IFITM proteins were under, we analyzed 9 primate IFITM1 or 9 primate IFITM3 sequences, including those of AGM we recently described [49] using Datamonkey [57–59] (Table 2) and MEGA6 software [44] (S1 Fig). In both cases, analysis of nucleotide sequences from IFITM1 and IFITM3 showed that the values of dN were much less than dS. Expanding the primate IFITM sequences to include additional IFITMs of other species from GenBank yielded similar results (data not shown), suggesting that IFITM proteins are under purifying selection (Table 2 and S1 Fig). Taken together, these results suggest that the primary amino acid sequence of the IFITM proteins are crucial for their biological functions and that most nonsynonymous changes could be deleterious to IFITMs.

**Table 2:** IFITM proteins undergo purifying selection.

	Codon	E[dS]	E[dN]	E[dN-dS]	Posterior Pr{dN>dS}	Bayes Factor {dN>dS}	Posterior Pr{dN<dS}	Bayes Factor {dN<dS}
**IFITM1**								
	5	2.48	0.22	-2.26	0	0	1	2196.89
	6	2.48	0.22	-2.26	0	0	1	2535.89
	7	2.48	0.22	-2.26	0	0	1	9062.38
	15	2.48	0.47	-2.01	0	0	1	1669.77
	19	2.48	0.22	-2.26	0	0	1	14377.9
	26	2.48	0.22	-2.26	0	0	1	10249.7
	51	2.48	0.22	-2.26	0	0	1	3188.27
	56	2.48	0.22	-2.27	0	0	1	121576000
	60	2.48	0.22	-2.26	0	0	1	16899.5
	71	2.48	0.22	-2.26	0	0	1	17067.2
	75	2.48	0.22	-2.27	0	0	1	16899
	81	2.48	0.22	-2.27	0	0	1	17727.4
	98	2.48	0.22	-2.26	0	0	1	25917600
	102	2.48	0.23	-2.25	0	0	1	11536.9
	105	2.48	0.22	-2.26	0	0	1	3956.29
	106	2.48	0.22	-2.26	0	0	1	1851.16
	118	2.44	0.46	-1.98	0.02	0.01	0.98	80.48
**IFITM3**								
	14	2.05	0.09	-1.96	0	0.01	1	108.21
	17	2.02	0.1	-1.92	0	0.02	1	59.42
	29	2.05	0.1	-1.95	0	0.01	1	101.33
	77	2.03	0.09	-1.95	0	0.01	1	84.3

Evidence of purifying selection in the primate IFITM proteins using the random-effects likelihood (REL) method assessed through the DataMonkey server (http://www.datamonkey.org). Only purifying selected sites are shown; no positive selected sites were detected. Purifying selected sites for IFITM1 and IFITM3 are indicated. A Bayes factors larger than 50 was considered significant. **Codon:** Indicates amino acid residue; **E[dS]:** Posterior mean of the synonymous substitution rate; **E[dN]:** Posterior mean of the non-synonymous substitution rate. **E[dN-dS]:** Posterior mean of the dN-dS difference; **Posterior Pr{dN>ds}:** Posterior probability for positive selection (dN>dS); **Bayes Factor {dN>dS}:** Bayes Factor (posterior odds/prior odds) for positive selection (dN>dS); **Posterior Pr{dN>ds}:** Posterior probability for negative selection (dN<dS); **Bayes Factor {dN>dS}:** Bayes Factor (posterior odds/prior odds) for negative selection (dN<dS).

## Discussion

In general, restriction factors are characterized with antiviral activity, are interferon inducible, targeted by viral antagonists, and undergo positive selection. Positive selection of restriction factors results from continual virus-host competition that occurs during infection. This effect is described by the Red Queen hypothesis, which results in an excess of non-synonymous mutations over time in both the virus and host restriction factors improving their overall fitness (positive selection). For example, the restriction factors TRIM5α, Tehterin, APOBEC3G, SAMHD1, and Viperin are all interferon inducible, restrict lentiviruses, and have undergone positive selection as a result of competing with viral antagonists [60–64].

IFITM proteins have been characterized as restriction factors as they are interferon inducible and restrict many enveloped viruses. Herein, we show that primate IFITMs have undergone purifying or negative selection suggesting that primate IFITMs are atypical restriction factors that are not directly antagonized by viruses. In this study, we determined the sequence for 14 new nonhuman primate IFITMs from Gorilla, L’Hoest’s, Moustached, Sykes, Mangabey, Mandrill, and Guereza and demonstrated that primate IFITMs have undergone purifying selection rather than positive selection. Negative selection of primate IFITMs would suggest they have not been highly antagonized by viruses; to date there are no known viral antagonists that directly target the IFITMs. These results may suggest that the primate IFITMs contain an ancient primary function that is not involved in viral restriction. Indeed, IFITMs have been implicated in several cellular functions including immunity, germ cell development, cell adhesion, oncogenesis, bone mineralization, proliferation and cell death [21, 65–68]. Furthermore, in the absence of IFN, IFITM proteins have been shown to be basally expressed in cells (including gorilla fibroblasts, Fig 6A) [65].

To determine if nonhuman primates conserved viral restriction, we tested their ability to restrict the primate lentiviruses HIV and SIV. Our observations show that nonhuman primate IFITMs are capable of restricting both HIV and SIV. Interestingly, some nonhuman primate IFITMs derived from OWMs appeared to be more efficient restricting both HIV and SIV compared to that of their human counterparts. For instance, IFITM3agm was more restrictive against SIVagm and HIV compared to human IFITM3, and this could be related to the conserved yet differential interactions between IFITM3 and primate lentivirus envelope proteins. These trends may imply that some adaptions of the primate lentiviruses have occurred although it is unclear whether these are direct or indirect methods of escape. Alternatively, IFITMs have been shown to contain additional functions such as altering membrane fluidity [19]. It may be that an alternative primary function of the IFITMs indirectly affects viral entry.

When challenged against two strains of HIV-1 (NL4.3 and BH10), human IFITM1 only marginally restricted HIV-1 NL4.3 (less than 2-fold) while it had a greater restriction against HIV-1 BH10 (greater than 3-fold). These observations are in close agreement with some earlier studies [15, 50]. Furthermore, we observed that nonhuman primate IFITM1 was capable of restricting both NL4.3 and BH10 (about 5- and 6-fold reductions, respectively, Fig 1), although they did not have interaction with HIV-1 Env (Fig 3). Interestingly, we found that IFTIM restriction against HIV-2 was nearly equivalent across all species of IFITM tested (about 2-fold reduction). This could suggest that certain strains of HIV-1 and HIV-2 have evolved to partially escape human IFITM restriction, though the mechanism(s) remain to be fully understood.

The IFITM proteins are capable of restricting viruses at various stages of infection [12, 13, 19, 29, 69]. We sought to determine if there was a particular stage during the HIV-1 life cycle in which nonhuman primate IFITMs inhibit HIV-1 compared to that of human IFITMs. Previously, our lab determined that human IFITMs restrict HIV-1 in part by antagonizing HIV-1 envelope [29]. In this work we found that interaction of envelope with OWM (L’Hoest’s) IFITM3 was conserved (Fig 3). However, only a slight increase (about 20%) in binding efficiency between OWM IFITM3 and HIV-1 envelope was observed (Fig 3). However, cell-to-cell transmission data clearly demonstrated that OWM IFITM3 is more efficient than human IFITM3 to reduce HIV-1 cell-to-cell spread and prolonged viral replication (Fig 5). These results together suggest that the main step of IFITM mediated restriction of HIV-1 occurs in the viral producer cells and is during cell-to-cell spread. It should be noted that the efficiency of HIV-1 cell-to-cell infection and replication in the presence of IFITMs are sometimes cell-type dependent [29].

While the various primate IFITMs are closely related and share a high degree of homology, there are several differences at the amino acid level that could account for stronger restrictive properties of some nonhuman primate IFITMs (S2 Fig). During purifying selection, changes that are deleterious to the gene function are removed from a population. In contrast, rare polymorphisms that present advantages to the population can occur and be preserved [70]. Though further investigation is needed, it is likely that polymorphisms in the IFITMs are responsible for their varying degrees of viral restriction [36, 71, 72]. It may also be that HIV/SIV Env has undergone positive selection that allows it to partially escape IFITM restriction to varying degrees [29, 51]. To date, no known viral accessory gene(s) have been identified to specifically antagonize the IFITM proteins [50], which could explain why primate IFITMs are under purifying selection. Hence, it is likely that the differential restrictions of IFITMs lie in the functional interactions between IFITM and primate lentivirus Env proteins. Indeed, we show here that human IFITMs are capable of interacting with HIV-1 envelope and that this interaction is evolutionarily conserved for OWM IFITM3 (Fig 3). More work is needed to elucidate the detailed mechanism(s) of IFITM restriction as well as viral countermeasures.

## Supporting Information

S1 Fig IFITM proteins are under purifying selection. (**a** and **b**) Phylogenetic trees of IFITM1 and IFITM3 genes were constructed using the Neighbor-Joining method and evolutionary distances were estimated using the Jukes-Cantor method. Bootstrap test percentages of 1000 replicates are shown next to the branches. (**c** and **d**) Selection analysis using the Nei-Gojobori method is shown. The probability of rejecting the null hypothesis dN = dS or rejecting dN = dS in favor of an alternative hypothesis is indicated. The test static is shown where the number of nonsynonymous and synonymous changes per site is represented by dN and dS, respectively. The bootstrap method using 1000 replicates was used to determine values. Analyses were conducted using MEGA6.
(TIF)

S2 Fig Amino acid sequence alignment of human and nonhuman primate IFITM proteins used in this study. Alignment of IFITM1 proteins (left) and IFITM3 proteins (right) is shown. Sequences were aligned using ClustalW.
(TIF)
