# Supplementary figures and images for: Nonhuman Primate IFITM Proteins Are Potent Inhibitors of HIV and SIV
Source: PLoS One. 2016 Jun 3;11(6):e0156739. doi: 10.1371/journal.pone.0156739 (PMC4892622; doi:10.1371/journal.pone.0156739)

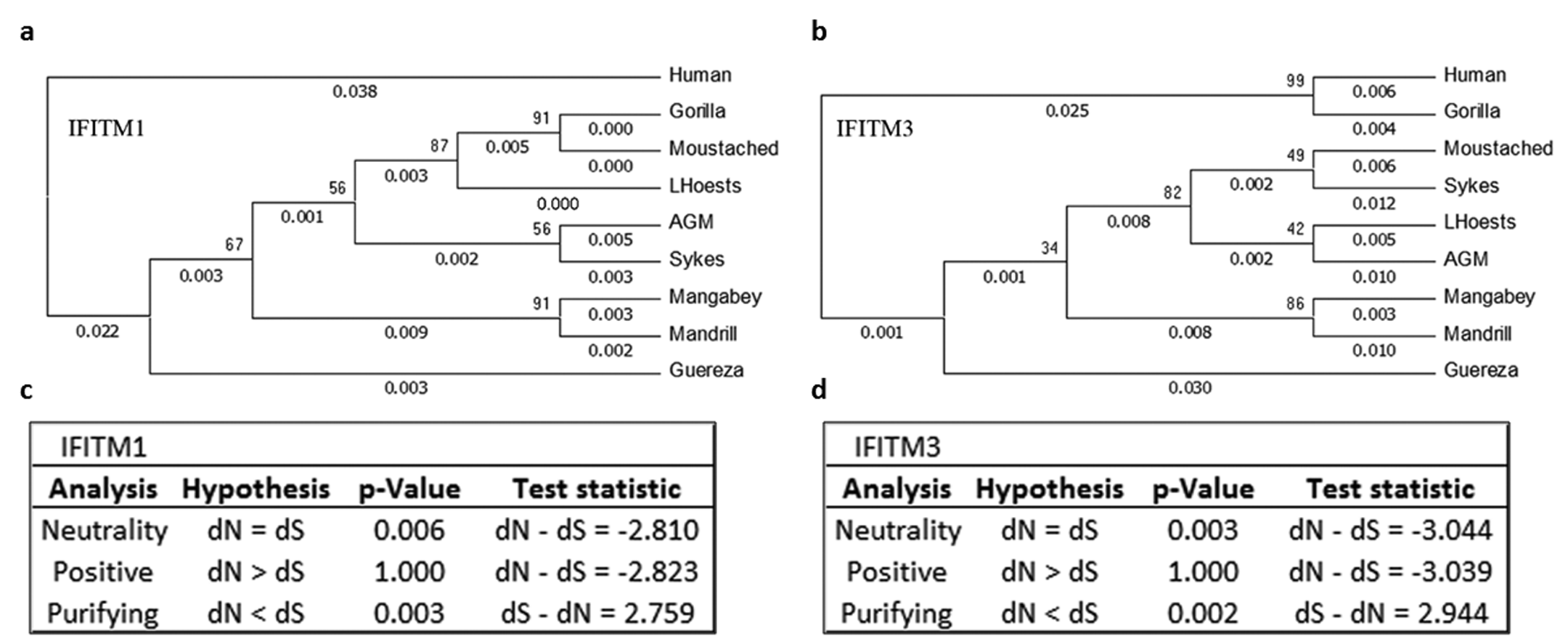

Supplement: S1 Fig — (a and b) Phylogenetic trees of IFITM1 and IFITM3 genes were constructed using the Neighbor-Joining method and evolutionary distances were estimated using the Jukes-Cantor method. Bootstrap test percentages of 1000 replicates are shown next to the branches. (c and d) Selection analysis using the Nei-Gojobori method is shown. The probability of rejecting the null hypothesis dN = dS or rejecting dN = dS in favor of an alternative hypothesis is indicated. The test static is shown where the number of nonsynonymous and synonymous changes per site is represented by dN and dS, respectively. The bootstrap method using 1000 replicates was used to determine values. Analyses were conducted using MEGA6. (TIF) [file pone.0156739.s001.tif]

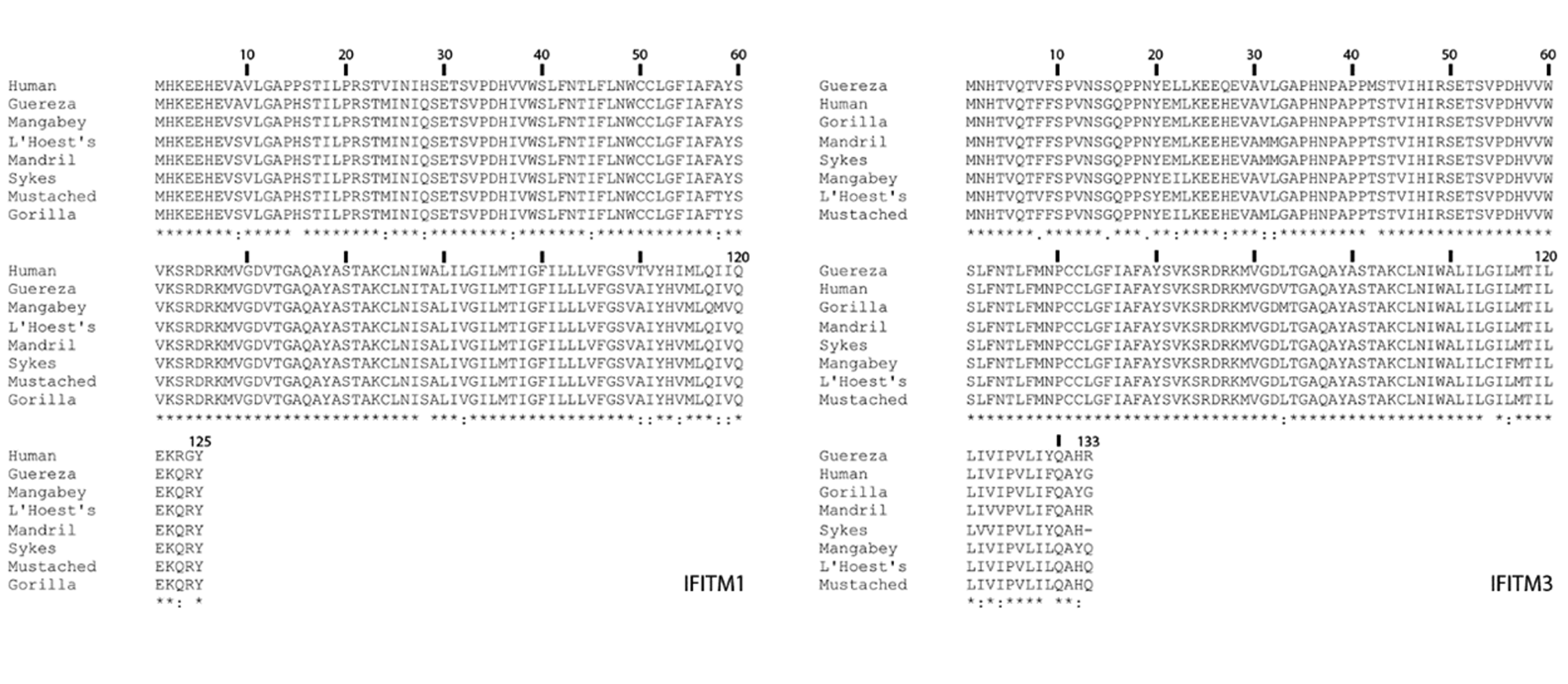

Supplement: S2 Fig — Alignment of IFITM1 proteins (left) and IFITM3 proteins (right) is shown. Sequences were aligned using ClustalW. (TIF) [file pone.0156739.s002.tif]
